# DC Electroacupuncture Effects on Scars and Sutures of a Patient with Postconcussion Pain

**DOI:** 10.1089/acu.2016.1188

**Published:** 2016-08-01

**Authors:** Antoine Chevalier, Kelly Armstrong, C. Norwood-Williams, Raman Gokal

**Affiliations:** ^1^ University of Natural Health, Sterling, VA.; ^2^ Women's Integrative Healing Inc., St. Augustine, FL.; ^3^ Necessity Strategies LLC, Melbourne, FL.

**Keywords:** Heart Rate Variability, Direct-Current Electroacupuncture, Postconcussion Syndrome, Headache Pain, Ataxic Tremors

## Abstract

**Introduction:** This case study offers a detailed comparative analysis of the effects of direct-current electroacupuncture (DC-EA) on the autonomic nervous system (ANS), when DC-EA was applied to the cranial sutures and scars of a patient with a history of ischemic stroke and postconcussion syndrome (PCS) pain.

**Case:** A 56-year-old female suffering from severe tremors and debilitating headaches requested acupuncture after conventional biomedicines failed to relieve her symptoms. Evaluations were performed to check the status of 27 ANS functions. These detailed evaluations were performed to obtain a baseline status of ANS function on this patient, who had a history of ischemic stroke, PCS, and chronic pain. All evaluations were repeated pre–post her DC-EA treatment.

**Results:** This patient experienced significant relief from her symptoms after DC-EA treatment. An analysis of this patient's risk for ANS complications showed improvements in four key homeostatic markers post treatment.

**Conclusions:** The ANS response of a patient with ischemic stroke, PCS, and chronic pain, who received electrical nerve stimulation using DC-EA reflected a measurable improvement in sympathetic tone, along with reductions in pain levels and PCS symptoms. The positive results in this case study could have applications to other pathologies that can be affected by the sympathetic nervous system activation on the body.

## Introduction

Postconcussion syndrome, also known as postconcussive syndrome or PCS, is a set of symptoms that might continue for weeks, months, or a year or more after a concussion—the latter being a minor form of traumatic brain injury.^1–3^ PCS symptoms include headaches, chronic pain, dizziness, fatigue, memory problems, and insomnia. The literature supports the concept that concussions from common traumas, as well as sports-related and military-related concussions, interfere with optimal levels of heart rate variability (HRV).^4–6^

According to the Centers for Disease Control and Prevention (CDC) there are up to 3.8 million concussions per year in the United States, with the incidence of sport-related concussions now reaching epidemic levels.^7^ The current standard for treatment of concussion is rest.^8^

It is widely accepted in science that imbalances of the parasympathetic (rest/healing/calming) and sympathetic (flight/fight/stress) branches of the autonomic nervous system (ANS) are directly linked to pain and a wide variety of and diseases.^9–15^ The sympathetic nervous system (SNS) is designed to facilitate short-term survival by creating a cascade of neurophysiologic responses. However, it is the upregulation or persistent tone in this system that is believed to be related to PCS symptomology.^6–8^

Microcurrent therapies involve applying weak direct currents (80 μA–1 mA) and are now being increasingly recognized as adjuncts for pain relief and ANS regulation.^16–20^

Microcurrent, applied transcranially, has been documented as being effective for treating anxiety, depression, insomnia, chronic pain,^21,22^ and, previously, for post-traumatic brain injuries,^23–25^ Microcurrent modulates cortical function and has been used to facilitate learning, alter behavioral performance, and improve impaired brain function.^26–31^

Studies of cranial sutures in adults have demonstrated a degree of movement, which contributes to the compliance and elasticity of the skull.^32^ Trauma to the head physically moves the cranial bones, stressing cranial sutures and affecting the ANS adversely in a similar manner as scars.^33^ Given that 80% of the fibers of the SNS go to the layers of the skin via the peripheral sympathetic fibers, these fibers' direct relationship to scars and cranial sutures is considered to be a major stressor of the ANS in patients who have PCS-related pain.

Scars and trauma have long been recognized in neural therapy as a source of ANS upregulation.^34–39^ It is theorized that damaged local cells lose their normal membrane potential, transmitting abnormal electric signals throughout the rest of the body via the ANS.^40,41^

This case study addresses how direct-current electroacupuncture (DC-EA) applied to sutures and scars influences ANS functioning and patient pain levels, when applied in a PCS treatment protocol. This is significant, as there is no consensus in the literature regarding the best practice for electrostimulation applied to patients with postconcussive symptomology. The purpose of this case report is to convey the effect of DC-EA on the ANS of a patient with symptoms of PCS.

## Case

### Presentation

A female client, G.G., suffering from severe tremors and debilitating headaches, presented for acupuncture treatment. G.G. was a 56-year-old, well-developed, well-nourished female in no apparent distress. Her weight was 72.3 kg, her height 170.2 cm, and her body mass index was 24.9.

G.G.'s medical history involved a major car crash, 25 years prior (1991), in which she reported having hit her head on a window and being diagnosed with a concussion. The severe headaches and tremors started soon after her sustained injury and were currently present most of the time but were accentuated with activity; this is also the presumed cause of her tremors. She had a previous surgical scar on her abdominal area from a Cesarean section in 1984, and a chest scar from a biopsy procedure in 1993. The severe headaches started after she suffered a thrombotic right cerebrovascular stroke 15 years prior in 2001. She had rehabilitative treatments for weakness in her left limbs and had no other disabilities.

She described having an aching pain at the bilateral frontal and corona capitis areas, with a Numeric Pain Rating Scale (NPRS) pain score of 7/10, and 10/10 on the “top of her head.”^39–41^ She wore a scarf over the top of her head because of the severity of pain she experienced when anything—even wind—touched the top of her head. Her cerebellar ataxic tremors were so severe that they interfered with writing and fine motor skills, such as pulling items out of her purse or wallet. Recently prior to presentation, she had received a medical suggestion from a physician to have a brain stimulation device implanted, with the hope of decreasing the hand tremors. She also reported having a tight and stiff neck.

She was opposed to medication and preferred to manage her diet and exercise regimen. She avoided both alcohol and caffeine and reported a having a consistent routine of standard mealtimes and sleeping habits. She had not been taking any prescription medications, choosing only ibuprofen to manage her headaches. Her physical examination revealed an ataxic tremor but no other abnormalities; the diagnosis she was given was PCS with ataxic tremors that occurred post–ischemic stroke. No magnetic resonance imaging or computed tomography scanning was performed for her case. To date, she has not had any specific treatment for these symptoms. She gave informed consent to receiving the DC-EA treatment.

### Treatment

DC-EA was applied simultaneously and bilaterally to acupuncture-trigger points located beside and along her cranial sutures and surgical scars using two^22,23^ Dolphin Neurostim (Acumed Medical LTD, Ontario, Canada) devices. These are U.S. Food and Drug Administration (FDA)–approved devices that are used to apply low-frequency, concentrated microcurrent stimulation (at 10K ohms) to relieve chronic pain.^22,23^ DC-EA application time was 30 seconds per point at approximately ½[[[inch mark]]]-intervals along the length of the scar-suture. As polarity of application is important, on one side of the scar-suture, the device was set to a negative pole (–) and on the other side of the scar-suture, the second device was set to a positive–negative pole (+/–). The intent of this methodology was to push a negatively charged current back and forth through a positively oriented suture or scar tissue. For this case study, all cranial sutures along with her two surgical scars, a transverse C-section scar, and a breast biopsy scar were treated. This treatment was applied once to this patient.

ANS assessments were taken before and after electrostimulation with an ANS1 (Biosensor Equipment LLC, Houston TX), an FDA-approved device that is used to measure HRV, sympathetic, parasympathetic, adrenergic, and cardiovagal functions. The device enables a multimodal approach to assessing SNS, parasympathetic nervous system, and galvanic skin response functions through an autonomic nerve assessment, an arterial assessment, and an assessment of cardiometabolic markers.^42,43^ Measurements of 27 physiologic “markers” are included to place each patient measurement categorically into abnormal, borderline, and optimal goal columns.

The Numeric Pain Rating Scale (NPRS) was used to evaluate this patient's pain. The NPRS is an 11-point scale from 0 to 10, with 0 being no pain and 10 being the most intense pain imaginable.^44–46^ The patient verbally selects a value that is most in line with the intensity of the pain that he or she has experienced in the last 24 hours, or the report will serve to rate pain experienced during a specific movement pattern or functional task. The NPRS has good sensitivity^46^ and excellent test–retest reliability.

The objective data collection was aimed at revealing:

(1) Whether or not DC-EA, when applied to sutures and scars, can modulate any variables within the ANS and scoring on the NPRS pain scale for a patient with PCS-related pain.
(2) Whether or not DC-EA is a valid option for nonpharmacologic pain management of PCS-related pain and symptoms.

## Results

G.G. reported a significant reduction in pain symptoms after treatment. Her initial report of a 7/10 severe baseline headache pain improved to “0” upon completion of the treatment and was reported during the post-test assessment. She also reported a 0/10 pain level when touching the top of her head and a loose and unrestricted neck. There was resolution of her presenting ataxic tremors. Six months later, at this writing, her headache and tremor symptoms have not returned, and she has not taken any headache medication since the DC-EA treatment (Table 1). Her neurologist commented to her that it appeared, according to her physical symptoms, that she was 93% improved from his baseline stroke assessment. Moreover, she has not taken any headache medication since the DC-EA application.

**Table 1.** G.G.'s NPRS Pain Score Pre- and Post-DC EA Treatment Showing Dramatic Improvement in All Scores

*NPRS Pain Scale*	*Pre–DC EA Treatment*	*Post–DC EA Treatment*
Headaches	7/10	0/10
Touch top of head	10/10	0/10
Tight/restricted neck	Tight	Loose/no restrictions
Severe ataxic tremors	Severe	None

NPRS, Numeric Pain Rating Scale, DC EA, direct current electroacupuncture.

### ANS Pre–Post Chart Results

Improvements were evident in all indicators of ANS function. The medical data related to the ANS improved, going from borderline into the optimal goal range.

The autonomic nerve assessment was focused upon four key homeostatic markers in the analysis of risk for ANS complications: (1) HRV, for which a straightforward and useful metric is the standard deviation of all normal R–R intervals (those measured between consecutive sinus beats on a Holter electrocardiogram); (2) the Stress Index; (3) Standard Deviation of All Normal to Normal (SDANN) results; and (4) a high-frequency (HF) indicator of parasympathetic vagal nerve activity. ANS dysfunction risk is based upon HRV analysis at rest, and it comprises ANS activity and balance of the SNS and ANS. Details on these markers is provided in the sections below.

### (1) HRV

**FIG. 1.**
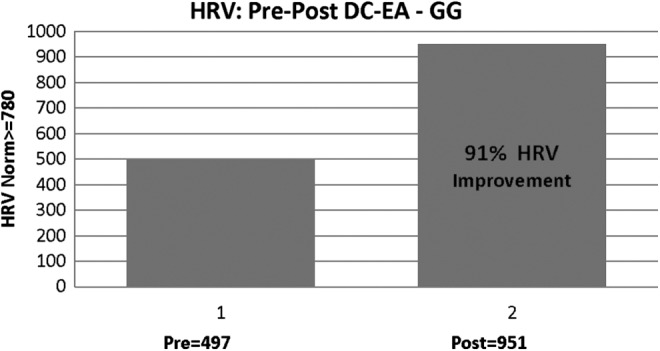
Patient G.G.'s heart rate variability (HRV)—total power. This parameter showed a marked (91%) improvement. HRV normal is ≥780 units. DC-EA, direct current electroacupuncture.

Total power has been determined to be the main indicator of ANS activity and is reflective of variations in time intervals between heartbeats, known as HRV. Lower than normal HRV values are associated with negative outcomes in heart disease and increased risk for diabetic neuropathy.^47^ The results of G.G.'s ANS evaluation, post application of DC-EA, showed distinct differences in outcomes (Fig. 1), with marked improvement in the HRV scores after DC stimulation was applied to the noted sutures-scars.

Low total power values could indicate a sedentary lifestyle and might indicate a need to increase physical activity. G.G.'s marked improvement in this value would indicate a significantly improved ANS response, compared to baseline and minimal target values, and should reflect an improvement in exercise tolerance and general health.

### (2) Stress Index

**FIG. 2.**
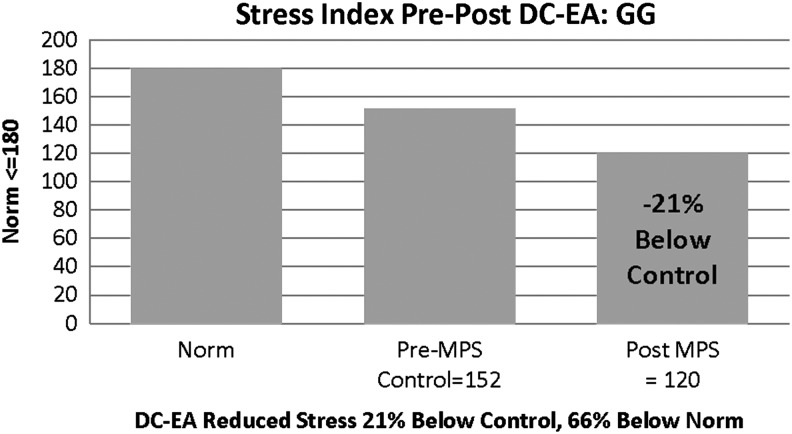
Patient G.G.'s Stress Index showed a 20% reduction post direct-current electroacupuncture (DC-EA), although the pre level was below normal (≤ 180 units) for this index.

This index is used to measure cardiac muscle oxygen demand related to heart work. The Stress Index is correlated with C-reactive protein (cRP) and is a marker of sympathetic failure.^48^ cRP is produced by the liver and increases with inflammation. HRV reflects the adaptability of the body to daily internal and external stressors that influence ANS function directly, as well as the stress the body is experiencing at the present time. High values indicate a risk for heart disease. Although G.G.'s values were below range to begin with, improvement for this indicator represented a marked improvement of her SNS (i.e., sympathetic downregulation), and subsequently, a reduction in her risk for heart disease if the improvement were to prove sustainable (Fig. 2).

### (3) SDANN

**FIG. 3.**
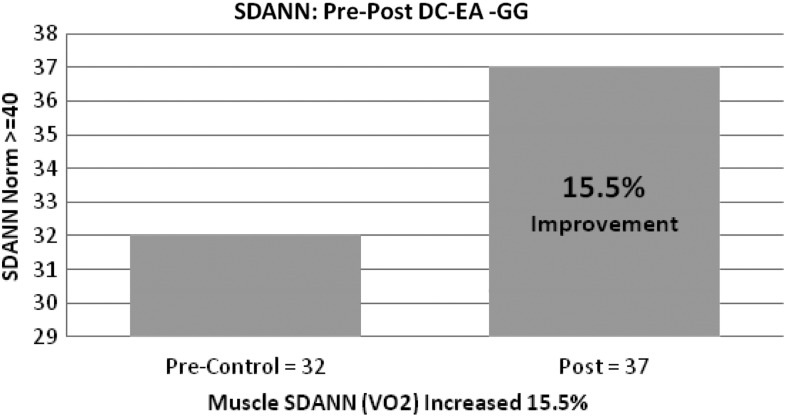
Patient G.G.'s Standard Deviation of All Normal to Normal (SDANN) showed a 15.5% improvement from 31 to 37 units. Normal SDANN is ≥40 units. DC-EA, direct current electroacupuncture.

The SDANN is a measure of ANS activity and V0_2_ (maximum oxygen consumption in the muscles).^49^ A low number indicates a sedentary lifestyle, with higher numbers primarily seen in athletes. Improvement in this marker would also imply an increased capacity for endurance, with a lower number reflecting the opposite response to physical activity. G.G.'s improvement in this indicator represents a marked downregulation and improvement of her SNS, as well as a subsequent increase in muscle oxygenation and exercise tolerance (Fig. 3).

### (4) HF Indicator of Parasympathetic Vagal Nerve Activity

**FIG. 4.**
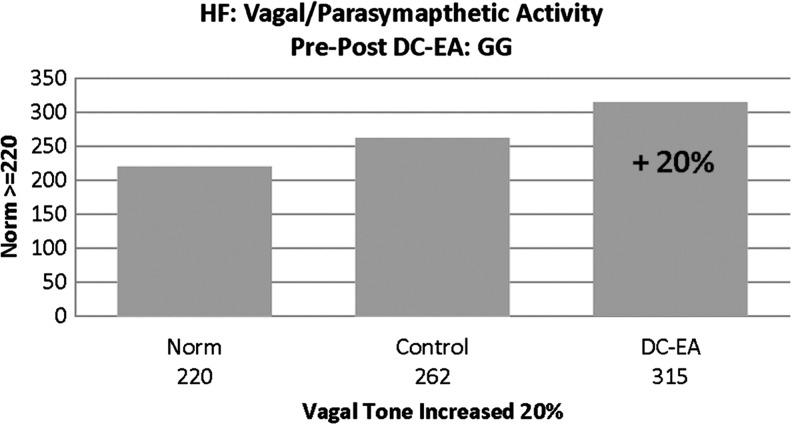
Patient G.G.'s high-frequency (HF) vagal/parasympathetic activity showed an increase of 20% with a normal value of ≥220 units. DC-EA, direct current electroacupuncture.

Vagal tone is an internal biologic process referring to the activity of the vagus nerve, which serves as the key component of the parasympathetic branch of the ANS. Research suggests that decreased vagal activity or tone is associated with increased stress vulnerability and poor health.^50^ A low value (< 220), suggests sympathetic system predominance and possibility of stress or mental anxiety. GG's baseline value for this indicator was below the established target for healthy ANS balance, yet improvement was reflected with DC-EA appearing to have an impact towards the goal of achieving optimal value (Fig. 4).

## Discussion

Sympathetic upregulation often results in stress and pain, which can make daily life miserable and lead to significantly impaired physical health.^51,52^ Both can be difficult to understand and, up to now, even harder to measure. Technology, such as advanced autonomic testing, can now provide real-time scientific evidence regarding the inner workings of human nervous systems in ill-health and disease,^53–55^ permitting collection of quantifiable data for the scientific study and education.

The data in this case study clearly showed that application of DC-EA improved the patient's health significantly. She reported severe headache (7/10), head pain (10/10), and ataxic tremors prior the DC-EA treatment, and her pain scores and hand tremors were reduced to nil (0) after DC-EA application to her suture-scars.

DC-EA also restored a more normal physiologic state throughout the patient's various nervous systems. There was marked improvement in her HRV Total Power score with DC-EA applied to acupuncture trigger points located beside sutures and scars. The patient's improvements in values would indicate a significantly improved ANS response, compared to baseline and minimal target values, and should reflect an improvement in exercise tolerance and general health. The improvement in the Stress Index represents a marked improvement of her SNS (i.e., downregulation) and, subsequently, a reduction in her risk for heart disease if the improvement were to persist. G.G.'s improvement in the SDANN suggests an improved tolerance to exercise. Her increased HF indicator of parasympathetic vagal nerve activity represents improved vagal–parasympathetic activity. Given that her pain and tremor symptomology have not returned 6 months post treatment, the positive outcomes of this case study suggest ANS regulation of PCS-related pain may help relieve PCS related symptoms.

It is suggested that a low-amplitude DC current mimics human biocellular communications, and its application may create a shift or change in cellular membrane configuration, producing a bodywide therapeutic effect. These biochemical processes might provide a plausible explanation for the prolonged autonomic modulation after the DC microcurrent; this is an area where future research is required.

## Conclusions

The current case study showed that DC-EA provided overall improvements in HRV, parasympathetic, and cardiometabolic markers, with subsequent long-term improvements in the patient's ataxic tremors and pain levels, suggesting a possible future role for DC-EA in the management of PCS, stroke, or stress-related diseases. Chronic PCS pain can limit quality of life, and restrict work and social engagement, and is often blamed for the development of drug dependencies of various forms. The changes produced in the ANS functions help validate the potential application of DC-EA to cranial sutures and scars as an option for clinicians treating patients with PCS-related chronic pain. However, further investigation is warranted with a much-larger focus group to confirm these results and to assess their duration.
